# Treatment of extranodal NK/T-cell lymphoma: From past to future

**DOI:** 10.3389/fimmu.2023.1088685

**Published:** 2023-02-07

**Authors:** Zheng Yan, Shuna Yao, Zhizhong Wang, Wenping Zhou, Zhihua Yao, Yanyan Liu

**Affiliations:** ^1^ Department of Internal Medicine, Affiliated Cancer Hospital of Zhengzhou University & Henan Cancer Hospital, Zhengzhou, Henan, China; ^2^ Department of Molecular Pathology, Affiliated Cancer Hospital of Zhengzhou University & Henan Cancer Hospital, Zhengzhou, Henan, China

**Keywords:** extranodal NK/T-cell lymphoma, asparaginase, PD-1/PD-L1 inhibitor, immunotherapy, treatment, novel drug, review

## Abstract

Extranodal NK/T-cell lymphoma (ENKTCL) is the most common subtype of T/NK-cell lymphoma in Asia and Latin America, but very rare in North American and Europe. Patient survival has improved significantly over the past two decades. However, standard treatment has not yet been established, although dozens of prospective trials have been conducted. To help understand how the treatment of ENKTCL has evolved in the past and what trends lie ahead, we have comprehensively reviewed the treatment of this aggressive malignancy, with a particular focus on neglected or unanswered issues, such as the optimal staging method, the best partner of asparaginase (Asp), the individualized administration of Asp, the preferred sequence of CT and RT and so on. Overall, the 5-year overall survival (OS) of patients with Ann Arbor stage I/II disease increased from < 50% in the early 20th century to > 80% in recent years, and the median OS of patients with Ann Arbor stage III/IV disease increased from < 1 year to more than 3 years. The improvement in patient survival is largely attributable to advances in radiation technology and the introduction of Asp and anti-PD-1/PD-L1 immunotherapy into practice. Radiotherapy is essential for patients with early-stage disease, while Asp-based chemotherapy (CT) and PD-1/PD-L1 inhibitors significantly improved the prognosis of patients with advanced-stage disease. ENKTCL management is trending toward simpler regimens, less toxicity, and higher efficacy. Novel drugs, such as manufactured T cells, monoclonal antibodies, and small molecule inhibitors, are being intensively investigated. Based on the fact that ENKTCL is highly resistant to cytotoxic drugs except Asp, and aggressive CT leads to higher toxicity rather than better outcomes, we recommend it is unnecessary to expend additional resources to compare different combinations of Asp with cytotoxic agents. Instead, more efforts should be made to optimize the use of Asp and immunotherapy to maximize efficacy and minimize toxicity, explore ways to overcome resistance to Asp and immunotherapy, identify novel treatment targets, and define subpopulations who may benefit more from specific treatments.

## 1 Introduction

Extranodal NK/T-cell lymphoma (ENKTCL) is a unique hematological malignant entity characterized by universal extranodal involvement and invariable Epstein-Barr virus (EBV) infection. The disease has a distinctive ethnic and geographic distribution. It is more common in East Asia and Latin America, but remarkably rare in other regions. ENKTCL is the most common T/NK-cell lymphoma subtype in Asia and Latin America. According to the latest data reported by the International Cooperative Non-Hodgkin T-cell Lymphoma Prospective Registry study (ICT study), ENKTCL accounted for 28.6% of T/NK-cell lymphomas in Asia during 2016-2019 (1). Relatively, the frequency is as high as 40-50% in Mexico (2, 3) and 8% in Europe and the United States (4).

ENKTCL most commonly originates from the mucosa of nasal cavity and adjacent structures, namely the upper aerodigestive tract (UAT) including the nasopharynx, oropharynx, oral cavity, and hypopharynx, resulting in destructive facial lesions. Other organs and tissues, such as skin, bones, digestive tract, lungs, liver, and reproductive organs, may also be involved in a small number of newly diagnosed cases and a large number of relapsed/refractory (R/R) cases. At presentation, most cases (70-90%) have UAT involvement and Ann Arbor stage I/II disease (1, 5–7).

Survival of patients with ENKTCL has improved significantly over the past two decades, and there is a consensus that for early-stage disease, radiotherapy (RT) is essential; for advanced-stage disease, Asp-based chemotherapy (CT) is superior to Asp-absent CT. Despite better patient outcomes and dozens of prospective studies, standard treatment has not been established. The reason is that most of these clinical trials had small sample sizes and focused on the efficacy evaluation of Asp in combination with different CT regimens. Further, many important questions remain unanswered, such as what is the best way to stage the disease? How to select patients with early-stage disease who do not require CT? How to optimize the efficacy of Asp-based CT? Which drug works best in combination with Asp? What is the optimal order for RT and CT? How many courses of CT are required for patients with early-stage disease? How to identify patients more likely to benefit from personalized treatment?

To help understand how treatments for ENKTCL have evolved in the past and where they may go in the future, and to help conduct well-designed clinical trials to accelerate the establishment of standard care, we comprehensively reviewed progress in the treatment of this disease over the past two decades. In particular, we mainly focused on several neglected or unanswered issues in the treatment of ENKTCL, including the optimal staging method, the best partner of Asp, the individualized administration of Asp, the preferred sequence of CT and RT and so on. The pathologic and genetic features of this disease have recently been well reviewed by others (8–10). When analyzing the outcomes of patients treated with different approaches or modalities, we visualized the data using figures, as most data are from retrospective studies with small cohorts and heterogenous treatments, which are difficult to compare directly.

## 2 Milestones in ENKTCL recognition and treatment

The condition of progressive necrotizing granuloma of the nasal cavity was first described in 1897, causing rapid invasion of the nose and face (midline) (11, 12). The disease was named in early literature as “malignant granuloma of nose”, “progressive lethal granulomatous ulceration of the nose”, “lethal midline granuloma”, and “granuloma gangraenescens” based on clinical characteristics (13), or “polymorphic reticulosis” and “angiocentric lymphoma” based on pathological characteristics (14). In 1982, it was recognized as a type of T-cell lymphoma (15). The association of this disease with EBV was first reported in 1985 (16). In 1987, The disease was identified as originating from NK cells (17). The revised European-American Lymphoma (REAL) classification, published in 1994, first presented this disease as a distinct subtype of malignant lymphoma called “angiocentric lymphoma” (18). A workshop in the same year comparing T-cell lymphoma in Asian and Western countries concluded that nasal T-/NK cell lymphoma, also called angiocentric lymphoma, is a distinct clinicopathologic entity. The workshop proposed nasal T-/NK cell lymphoma for midline facial lesions and nasal-type T-/NK cell lymphoma for tumors in other anatomic sites (19). The 2001 WHO classification named this entity as “extranodal NK/T-cell lymphoma, nasal type” (20) and later, in the 5^th^ edition of the WHO classification of haematolymphoid tumors in 2022, “extranodal NK/T-cell lymphoma, nasal type” was renamed “extranodal NK/T-cell lymphoma”.

**Figure 1:**
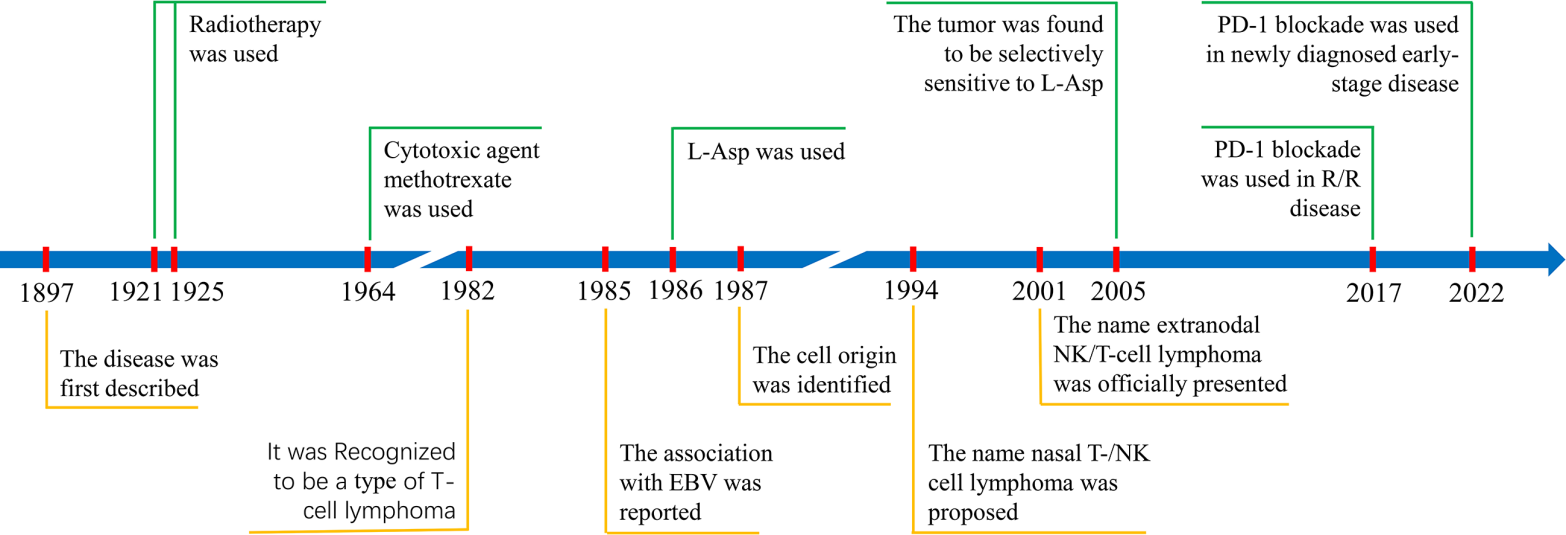
Milestones in the recognition (shown in yellow below the timeline) and treatment (shown in green above the timeline) of ENKTCL.

Before the 1920s, surgery, antibiotics, and steroids had been used to treat ENKTCL, but all had failed. Durable responses were not observed until the use of RT (radium implantation in 1921 and X-rays in 1925) (12, 21, 22). Since then, RT has been increasingly used in practice. For CT, responses were anecdotally observed in patients treated with the antimetabolite methotrexate in 1964 (23). However, CT has been an adjuvant therapy for a long time due to its low response rate and rapid development of drug resistance. This state persisted until the introduction of L-asparaginase (L-Asp). The first case of L-Asp-induced complete response (CR) in refractory ENKTCL was reported in 1986 (24). In 2005, ENKTCL cells were found to be selectively sensitive to L-Asp (25), but long-term survivors were still rare in patients with advanced-stage disease until the introduction of programmed cell death protein 1 (PD-1) and its ligand (PD-L1) blockade immunotherapy in recent years. PD-1 blockade was reported to be highly active in relapsed/refractory (R/R) ENKTCL patients in 2017 (26) and later used to treat newly diagnosed early-stage disease in 2022 (27). The milestones in the recognition and treatment of ENKTCL are summarized in **Figure 1**.

## 3 Survival trend of patients with ENKTCL in the past two decades

**Figure 2:**
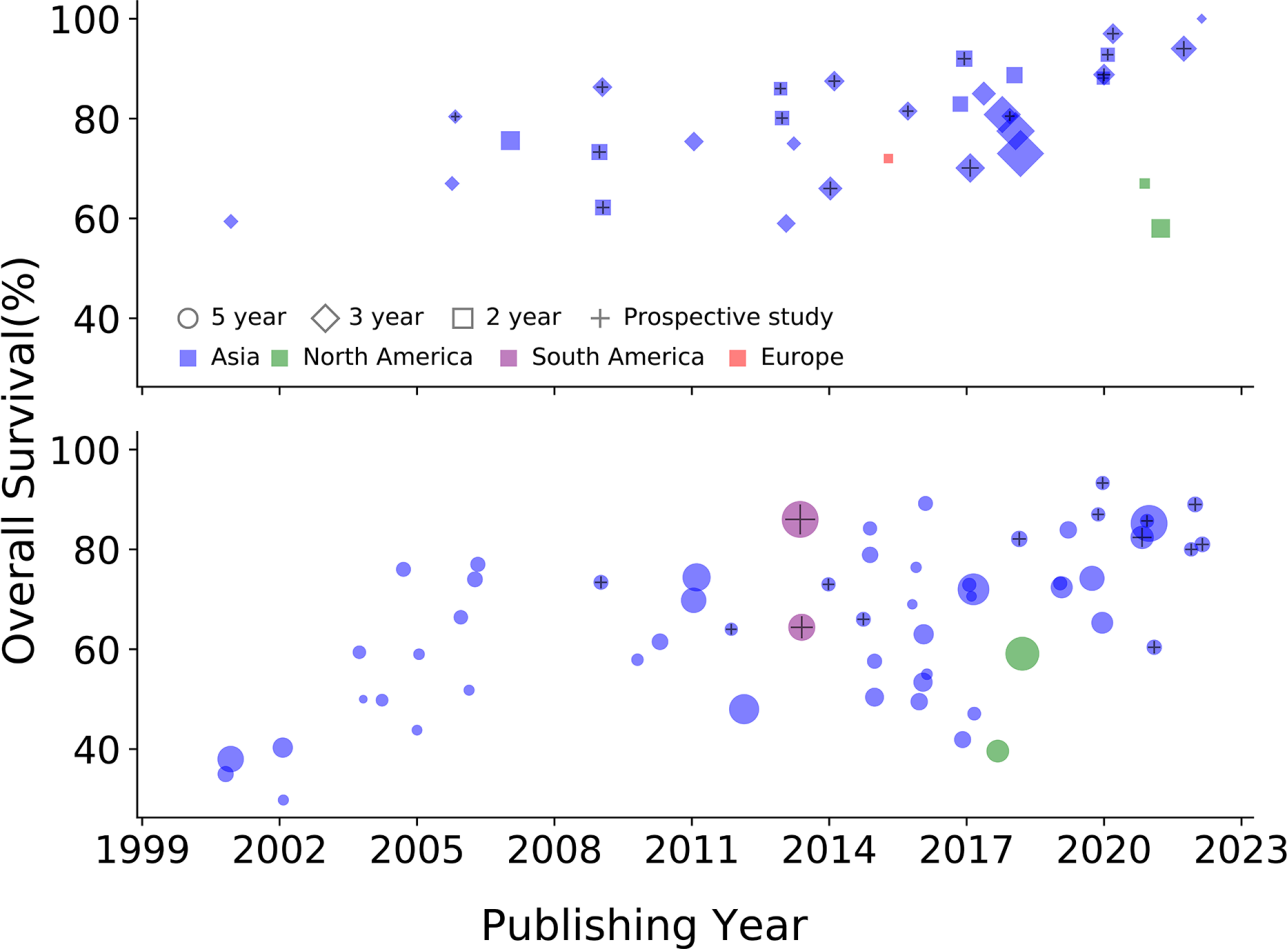
Survival trends of patients with Ann Arbor stage I/II ENKTCL over the past two decades. Only data from patients treated with RT or RT plus CT are included. The area of each marker in the figure indicates the sample size of the study. Data are cited from (27–97).

**Figure 3:**
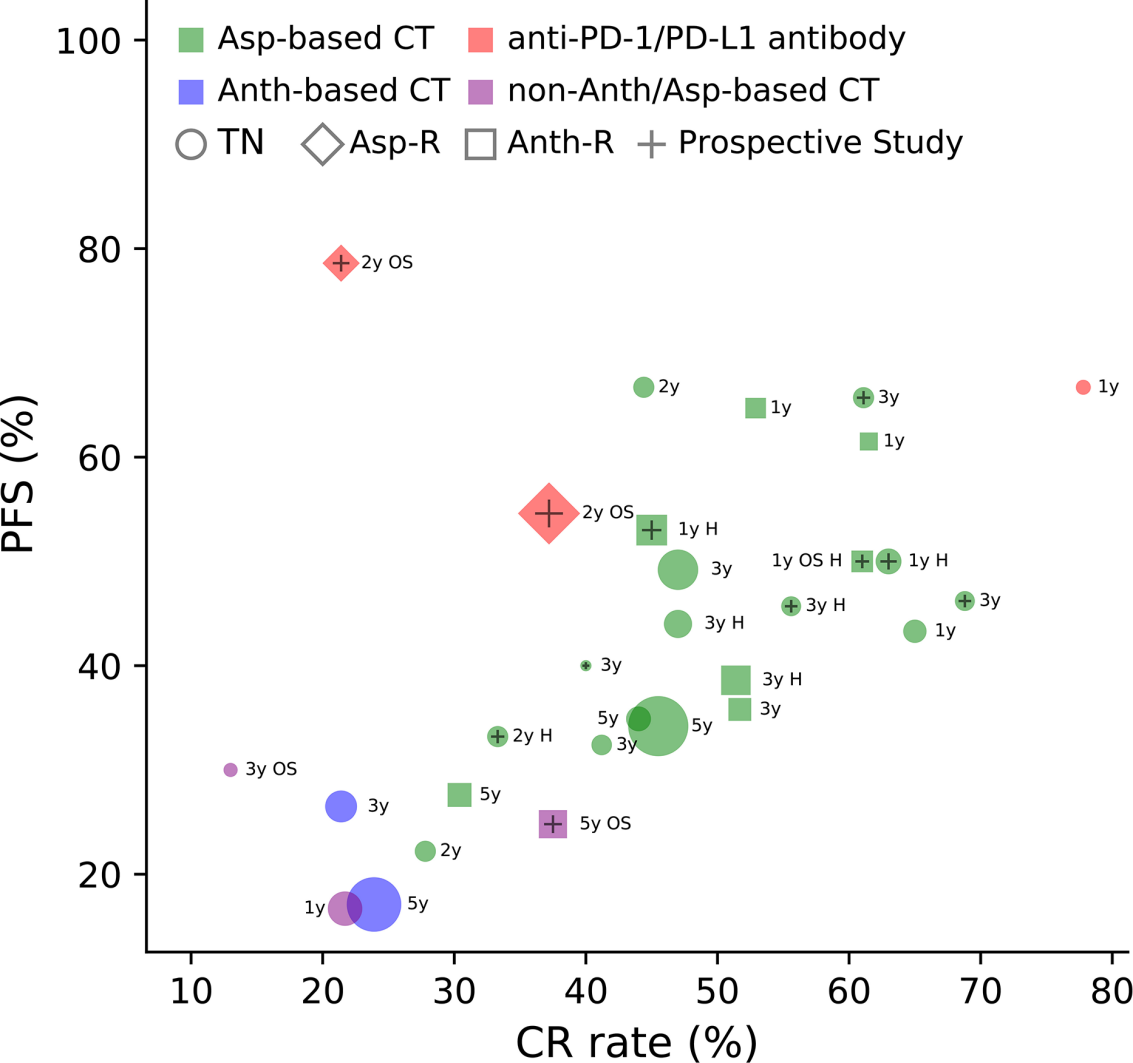
Complete response (CR) rate and progression-free survival (PFS) of patients with Ann Arbor stage III/IV or relapsed/refractory ENKTCL treated with different drug types. Asp, asparaginase; Asp-R, asparaginase-resistant; Anth, anthracycline; Anth-R, anthracycline-resistant; CT, chemotherapy; TN, treatment naïve; OS, overall survival (when PFS is not available, OS is displayed); y, year; H, hematopoietic stem cell transplantation. The area of each marker in the figure indicates the sample size of the study. Data are cited from (35, 68, 69, 72, 73, 82, 84, 94, 98–114).

ENKTCL is one of the tumors with the most dramatic change in survival over the past 20 years. The 5-year overall survival (OS) for patients with Ann Arbor stage I/II disease has increased from < 50% in the early 2000s to > 80% in recent years. Survival data for early-stage disease published since 2000 are summarized in **Figure 2**. Here, we included only patients treated with RT or RT plus CT because RT is essential for early-stage disease. The improved survival can be attributed to several factors: the accumulation of knowledge about the biological behavior of the disease, accurate staging with modern photographic techniques, advances in RT techniques, and the introduction of Asp-based CT. Significant changes in survival were also seen in patients with advanced-stage disease. The median OS for advanced-stage disease has increased from a few months to more than 3 years. The better prognosis in advanced-stage disease is largely attributable to the introduction of Asp and PD-1/PD-L1 blockade immunotherapy. Data of advanced-stage disease published since 2000 are summarized in **Figure 3**.

## 4 The role of Asp in ENKTCL treatment

### 4.1 Asp is a game changer

ENKTCL is known to be resistant to conventional CT. In most reports, the CR rate in patients treated with anthracycline (Anth)-based CT was around 30% (29, 43, 46, 93). The resistance of ENKTCL to multiple cytotoxic drugs was initially attributed to the expression of P-glycoproteins (P-gp) on tumor cells (115). However, ENKTCL is also extremely resistant to P-gp independent agents such as methotrexate and cytarabine (25), and the response and survival of patients treated with P-gp independent agents are disappointing. In Anth-resistant ENKTCL patients treated with gemcitabine-based CT, the CR rate and median progression-free survival (PFS) were only 20% and 2.3 months, respectively (116). Further, in two prospective studies, the CR rates in patients with stage III/IV or Anth-resistant disease treated with the IMEP regimen (ifosfamide, methotrexate, etoposide, and prednisone) were 8% and 32%, respectively (35, 99). The absolute efficacy of CT is clearly demonstrated in patients with advanced-stage disease (**Figure 3**). We can see that the efficacy of non-Anth/Asp-based CT is as poor as the efficacy of Anth-based CT.

However, CR rates with Asp-based CT range from 40% to 70% in most reports, both in early-stage or advanced-stage disease, and in treatment naive or Anth-resistant disease (**Figure 3**). A multicenter retrospective study from the China Lymphoma Collaborative Group analyzed data from 286 newly diagnosed advanced-stage ENKTCL cases and found that patients treated with Asp-containing CT had a significantly higher CR rate (45.5% *vs*. 23.9%, *P* = 0.006), 5-year PFS (34.2% *vs*. 17.1%, *P* < 0.001), and OS (45.3% *vs*. 27.8%, *P* < 0.001) than those treated with Asp-absent CT (110). The superiority of Asp-based CT over non-Asp-based CT is convincing enough through historical comparison, despite the lack of randomized controlled trials (**Figure 3**). This conclusion is also supported by systemic reviews and meta-analyses (117).

### 4.2 Anti-lymphoma mechanism of Asp

**Figure 4:**
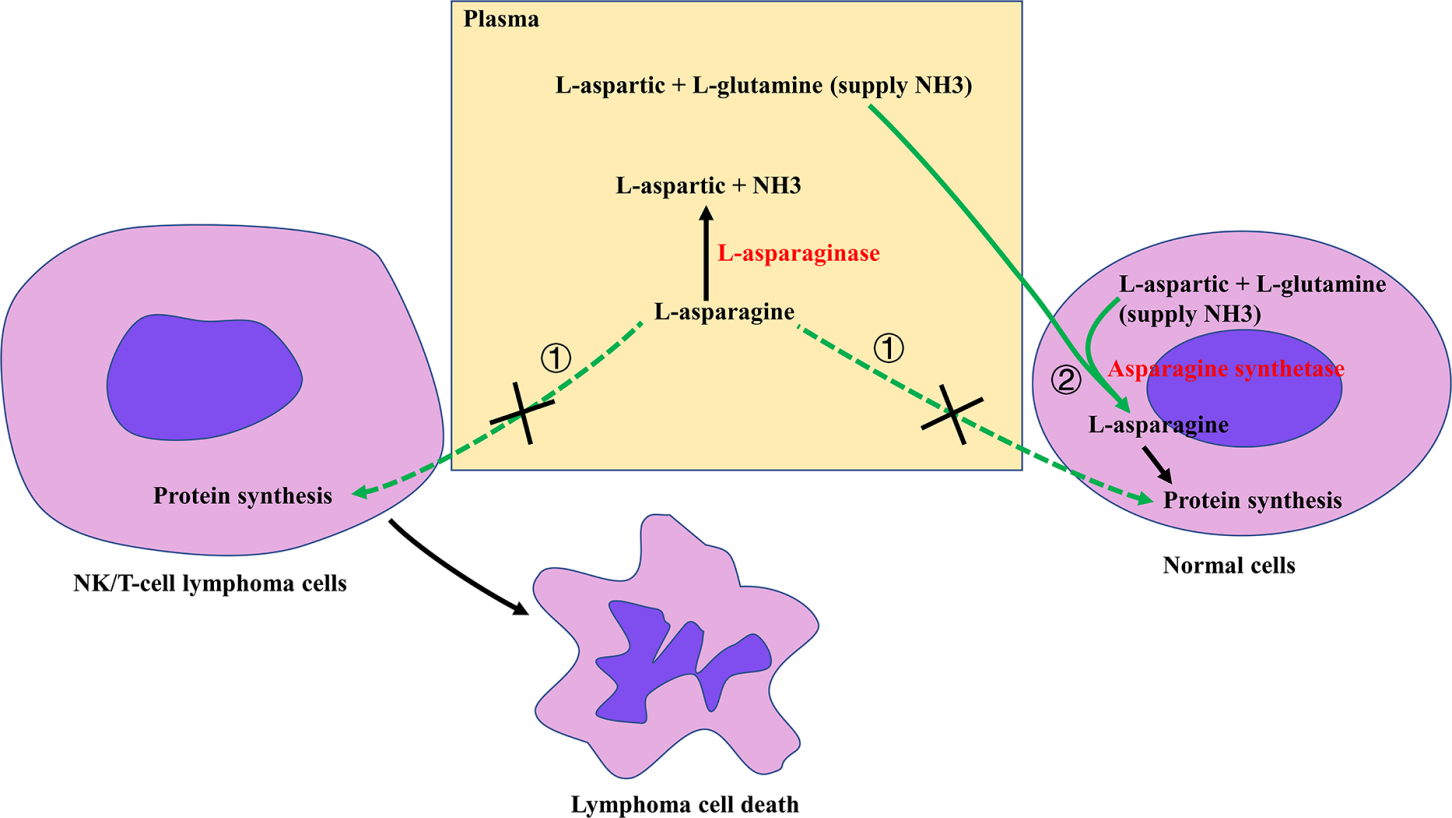
Overview of the mechanism of action of asparaginase (Asp) against NK/T-cell lymphoma (NKTCL). Due to the lack of asparagine synthetase (AsnS), NKTCL cells depend on the uptake of extracellular L-asparagine from the circulation (pathway ①) for protein synthesis. Administration of Asp hydrolyzes serum asparagine to aspartic acid and ammonia (NH3), thereby inhibiting tumor cell protein synthesis and ultimately leading to tumor cell death. In contrast, all normal cells in the body have two pathways to obtain L-asparagine for protein synthesis: taking extracellular L-asparagine from the circulation (pathway ①) and synthesizing their own L-asparagine from aspartic acid and NH3 *via* AsnS (pathway ②). When pathway ① is blocked, normal cells can still obtain adequate L-asparagine *via* pathway ② to meet their metabolic needs.

The excellent anti-ENKTCL activity of Asp is attributed to its unique anti-tumor mechanism and the natural weakness of ENKTCL cells. Asparagine is a non-essential amino acid that can be synthesized enzymatically from aspartic acid and ammonia in all normal cells of our body in the presence of asparagine synthetase (AsnS). L-Asp, in contrast to AsnS, is an enzyme that selectively hydrolyzes the extracellular amino acid L-asparagine to L-aspartate and ammonia. L-Asp has been introduced for the treatment of childhood acute lymphoblastic leukemia (ALL) since the 1960s because ALL cells lack AsnS expression and depend on extracellular asparagine uptake for survival. When plasma asparagine is depleted by systemic L-Asp administration, intracellular protein biosynthesis ceases and ALL cells die. The low expression of AsnS in ALL cells is due to hypermethylation of the AsnS gene. The CpG island methylation status of the AsnS gene promoter is associated with Asp sensitivity and its hypomethylation status correlates with Asp resistance and is an adverse prognosticator of patient survival (118). ENKTCL cells are even more sensitive to L-Asp than ALL cells (25). L-Asp was first used in combination treatment of ENKTCL by Murase et al. in 1986 (24). Around 2000, several case reports showed that L-Asp monotherapy induced durable CR in patients with R/R ENKTCL (119–121). Yong et al. reported a cohort of 18 (7 early-stage and 11 advanced-stage) Anth-resistant ENKTCL patients treated with L-Asp, vincristine, and dexamethasone followed by RT (if applicable). The CR rate after CT was 55.6%, and the 5-year OS rate of the whole cohort was 55.6% (122). Subsequent *in vivo* studies by Ando et al. showed that NK-cell leukemia/lymphoma cell lines were selectively sensitive to L-Asp among multiple tested anti-cancer agents. Further examination of tumor samples revealed that AsnS mRNA expression levels in lymphoma cells were inversely corelated with clinical responses to L-Asp treatment (25). These findings were subsequently confirmed by other studies (123, 124). Obviously, ENKTCL has the similar biological property to ALL (**Figure 4**). Likewise, the low expression of AsnS gene in ENKTCL cells may be due to genome-wide DNA hypermethylation (125).

### 4.3 L-Asp and pegaspargase

The use of Asp in the treatment of ALL has been intensively studied. Its optimal therapeutic effect depends on complete and sustained depletion of serum asparagine (126). The serum half-life (t_1/2_) of native *E. coli* L-Asp is 1.25 days (127). When using native Asp, daily administration is unnecessary. Instead, it can be administered at 2- to 3-day intervals. It is crucial, however, that the drug should be administered throughout the treatment period to achieve a complete and sustained depletion of serum asparagine (126). If L-Asp is administered for 4 or 7 days at 3-week intervals, starved cancer cells may be revived by regaining asparagine during the Asp-absent intervals. This may explain, at least in part, the unsatisfactory outcomes in some studies using L-Asp. Currently, L-Asp is largely replaced by polyethylene glycol-conjugated Asp (pegaspargase, PEG-Asp). The serum t_1/2_ of PEG-Asp is 5.73 days, making it a 2-week dosing interval (127). The incidence of hypersensitivity to PEG-Asp is lower than that to native L-Asp (128).

**Figure 5:**
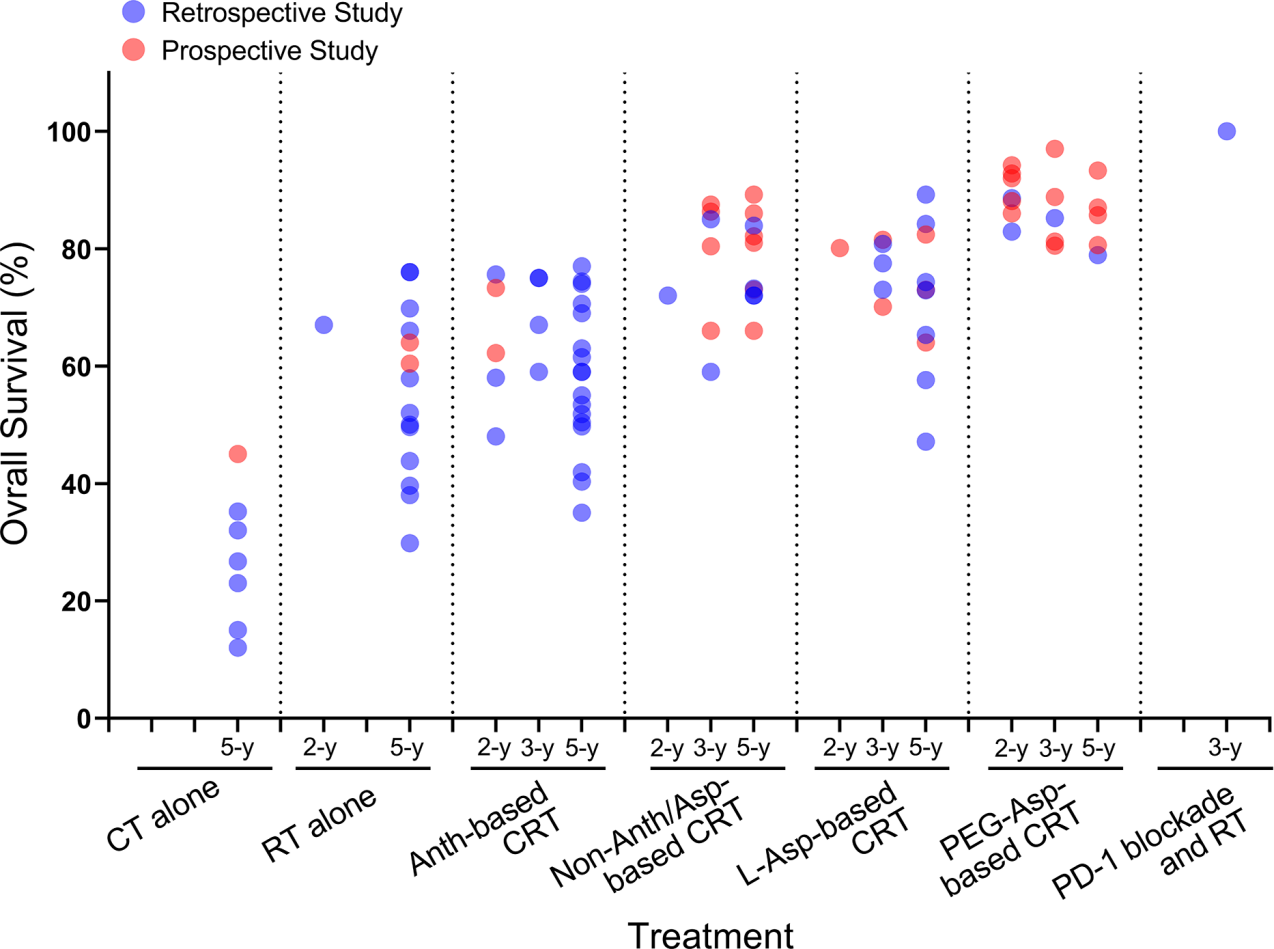
OS trends in early-stage ENKTCL patients treated with different modalities. CT, chemotherapy; RT, radiotherapy; Anth, anthracycline; CRT, chemoradiotherapy; Asp, asparaginase; PEG-Asp, pegaspargase; OS, overall survival. Data cited from (4, 27–32, 34–37, 39, 40, 42–46, 48–82, 84–97).

Theoretically, both forms of Asp have similar anticancer activity, but in practice, L-Asp is inferior to PEG-Asp (**Figure 5**). Part of solid evidence comes from two prospective studies comparing L-Asp with PEG-Asp in patients with ENKTCL. One study by Kim et al. compared L-Asp *vs*. PEG-Asp in combination with the IMEP regimen in 41 newly diagnosed stage IV or Anth-resistant ENKTCL patients. L-Asp was delivered on days 1, 3, 5, 7, 9, and 11 of each cycle of CT, and PEG-Asp was given every 3 weeks. The results showed that the CR rate was higher in the PEG-Asp group (73.7% *vs*. 45.5%; *P* = 0.067) (129). In another recent randomized controlled study, 80 newly diagnosed ENKTCL patients were assigned to the DDGP (dexamethasone, cisplatin, gemcitabine, and PEG-Asp, repeated at 3-week intervals) arm or the SMILE (dexamethasone, methotrexate, ifosfamide, etoposide, and L-Asp, L-Asp given at 3-week intervals on days 3 to 9) arm. The results showed that the 3-year PFS (56.6% *vs*. 41.8%, *P* = 0.004) and 5-year OS (74.3% *vs*. 51.7%, *P* = 0.02) in the DDGP arm were significantly higher than those in the SMILE arm (130). L-Asp-based CT was inferior to PEG-Asp-based CT, at least in part due to the long L-Asp dosing interval between two courses of CT. Besides, higher toxicity-related mortality in the second study was also responsible for the poorer outcome in the SMILE arm (17.5% in the SMILE arm *vs*. 2.5% in the DDGP arm).

### 4.4 Individualized administration of Asp

The efficacy of Asp-based CT will be further enhanced if individualized treatment is given. The main disadvantages of Asp are hypersensitivity and silent inactivation, both of which are attributed to the production of neutralizing anti-Asp antibodies, occurring in 30-70% of patients during L-Asp treatment (131). Silent inactivation refers to the phenomenon of neutralizing antibodies being present in the absence of any clinical signs of allergy. Anti-Asp antibodies may result in faster Asp clearance, lower serum Asp concentrations, and higher risk of relapse (132, 133). PEG-Asp should not be given to patients who develop anti-*E. coli* Asp antibodies. Instead, Erwinia L-Asp which has different antigenic epitope to *E. coli* Asp is an alternative (134). For PEG-Asp, in addition to anti-Asp antibodies, there is another problem. Anti-PEG antibodies produced after PEG-Asp treatment can also lead to rapid Asp clearance and reduced efficacy (135). Moreover, Asp is essentially a protein that may have significantly different pharmacokinetics, pharmacodynamics, and immunogenicity in patients of different races and ages. For example, the t_1/2_ of PEG-Asp is 7.1 days in South American adults and 5.73 days in Caucasian children (127, 136). Therefore, it is better to monitor the efficiency of asparagine depletion during treatment to achieve optimal efficacy and avoid ineffective medication.

**Figure 6:**
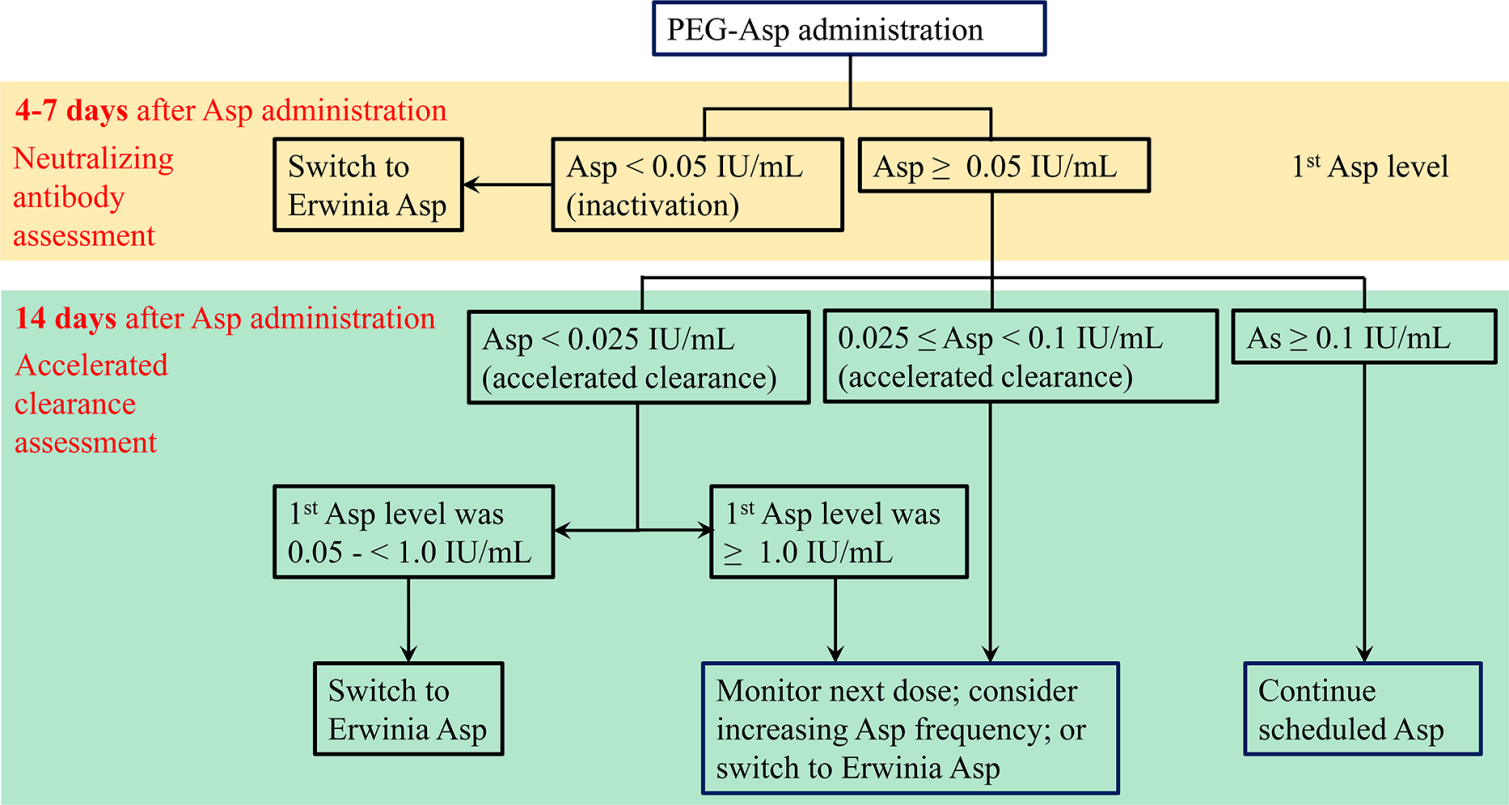
PEG-Asp activity monitoring algorithm for assessing possible silent inactivation by neutralizing antibodies and/or accelerated clearance (137).

Asp depletion efficiency can be evaluated by serum Asp concentration, presence of anti-Asp antibodies, and Asp activity. Relatively, Asp activity assay remains the best option in practice due to the lack of an anti-Asp antibody test that differentiates between inactivating or non-inactivating antibodies and the rapid ex vivo metabolism of asparagine in the presence of Asp (131). Currently, in the absence of detailed data on Asp metabolism in adult ENKTCL patients, the PEG-Asp activity monitoring algorithm proposed by Archie et al. can be adopted to optimize PEG-Asp dosing (137). Briefly, the algorithm consists of two steps: the first Asp activity monitoring is performed 4 - 7 days after PEG-Asp administration. Asp activity < 0.05 IU/mL indicates the presence of neutralizing antibodies, so treatment should be switched to Erwinia Asp. On the other hand, if Asp activity is ≥ 0.05 IU/mL, a second Asp activity monitoring should be performed 2 weeks after PEG-Asp administration. A second test result of < 0.025 IU/mL and a first test result of < 1.0 IU/mL indicate the presence of accelerated clearance, so the treatment should be switched to Erwinia Asp. If the second test result is < 0.025 IU/mL and the first test result is ≥ 1.0 IU/mL, or the second test level is between 0.025 and 0.1 IU/mL, there are three options: monitoring Asp activity after next dose, increasing PEG-Asp frequency, or switching to Erwinia Asp. If the second test level is ≥ 0.1 IU/mL, the scheduled PEG-Asp administration can be continued (**Figure 6**). For ENKTCL, if silent inactivation or hypersensitivity occurs and Erwinia L-Asp is not available, RT (for early-stage disease) or second-line treatment (for advance-stage disease) should be moved forward.

In addition, the dose of PEG-Asp used in current practice may not be optimal. As Asp is an enzyme, when the substrate (asparagine) is saturated, the pharmacological effects (both efficacy and toxicity) of Asp do not change with increasing dose. This has been confirmed in practice. Kloons et al. conducted a trial to evaluate individualized dosing of PEG-Asp in pediatric ALL patients. They demonstrated that a median dose of 450 IU/m^2^ PEG-Asp was enough to achieve adequate Asp activity levels and sufficient asparagine depletion, and that the reduced dose of PEG-Asp had similar toxicity to the standard dose of PEG-Asp (138). More importantly, the frequency of Asp treatment matters. As noted above, optimal therapeutic efficacy of Asp depends on complete and sustained depletion of serum asparagine. When used in combination with other cytotoxic agents, PEG-Asp is usually repeated every 3 weeks. However, approximately 20% of patients are unable to maintain adequate levels of Asp activity in the third week of each 3-week interval (136), so PEG-Asp should be repeated every two weeks if Asp activity levels are not monitored (139).

### 4.5 Best partner of Asp

It seems that it does not matter which drugs are combined with Asp in combination therapy. On the one hand, ENKTCL is intrinsically resistant to cytotoxic agents other than Asp. On the other hand, there is no evidence that one combination is more favorable than the other, regardless of toxicity. To date, two randomized controlled studies have comparatively evaluated PEG-Asp-based regimens for ENKTCL, and both obtained similar efficacy across treatment arms (**Table 1**). Huang et al. reported preliminary results of P-GemOx (PEG-Asp, gemcitabine, and oxaliplatin) + thalidomide *vs*. AspMetDex (PEG-Asp, methotrexate, and dexamethasone) at the 2019 ASH conference. They observed similar CR rates (60.0% *vs*. 55.0%), 3-year PFS and OS (detailed data not reported) between the two arms of 165 patients with newly diagnosed or R/R disease (143). Wei et al. compared SVILE (ifosfamide, PEG-Asp, vindesine, etoposide, and dexamethasone) with P-GemOx in 103 treatment-naïve patients. The CR rates after three cycles of CT (38.9% *vs*. 39.4% for early-stage disease, *P* = 0.789; 6.2% *vs*. 11.1% for advanced-stage disease, *P* = 1.000), 3-year PFS (88.3% *vs*. 93.9% for early-stage disease, *P* = 0.469; 46.2% *vs*. 65.7% for advanced-stage disease, *P* = 0.703), and 3-year OS (88.8% *vs*. 97.0% for early-stage disease, *P* = 0.130; 68.8% *vs*. 72.2% for advanced-stage disease, *P* = 0.729) were all similar between the two arms (69). In addition, Wang et al. treated 30 patients with PEG-Asp alone in a prospective phase 2 study. Two cycles of PEG-Asp were delivered concurrently with RT and 4 more cycles after RT. The results were excellent, with 2-year PFS of 90.9% and OS of 92.8% (67). Obviously, a more simplified regimen is more favorable when drug toxicity is considered. While this does not mean that those partners of the Asp scheme are of no values. A positive thought about Asp partners is that ENKTCL cells become sensitive to these partners when they are starved for asparagine deficiency following Asp administration. Based on the fact that aggressive chemotherapies result in higher toxicities rather than better efficacy, we can conclude that the highly toxic partners are unnecessary when combined with Asp. We suggest that more resource-consuming comparisons of Asp combination with different cytotoxic agents are unnecessary without the introduction of innovative concepts.

**Table 1:** Staging systems for ENKTCL.

Staging system	Staging rule	Advantage	Disadvantage
Ann Arbor (140)	Stage I, single extranodal lesion without nodal involvement; stage II, stage I or II nodal extent with limited contiguous extranodal involvement; stage IV, additional non-contiguous extranodal involvement	Easy to use	Stage I includes highly heterogenous disease due to varying extent of LTI
Korea (141)	Limited disease: Ann Arbor stage I/II UAT disease without LTI; extensive disease: Ann Arbor stage I/II UAT disease with LTI or stage III/IV UAT disease, and non-UAT disease.	Easy to use	Both limited and extensive diseases include highly heterogenous disease
TNM (7)	T stage is based on the anatomical structures involved; N stage is based on the extent of RLNM; all lesions beyond the UAT and regional lymph nodes are defined as M1.	Tumor burden and survival risk stratified well.	Only UAT disease is included; too complex and inconvenient for use
CA (142)	Stage I, confined to nasal cavity or nasopharynx without LTI and RLNM; stage II, non-UAT disease or UAT disease with LTI, without RLNM; stage III, disease with RLNM; stage IV, non-RLNM or disseminated disease	Easy to use; Stage I, III, and IV reflect tumor burden and survival risk well	Stage II includes heterogenous disease due to varying extent of LTI.

LTI, local tumor involvement; UAT, upper aerodigestive tract; RLNM, regional lymph node metastasis; CA, the Chinese Southwest Oncology Group and Asia Lymphoma Study Group.

## 5 Staging methods for ENKTCL

Staging is very important for ENKTCL because of its solid cancer features: the majority of patients present with early-stage disease and RT is a curative treatment; the extent of local tumor invasiveness (LTI) is the strongest prognosticator in patients with early-stage disease (7, 31, 144). It is believed that RT alone is sufficient for patients with very early-stage disease. However, there is no consensus on how to define very early-stage disease. Future randomized controlled trials may answer this question, but an appropriate staging method is a prerequisite.

The Ann Arbor system is by far the conventional and most widely used staging method. This staging method was originally designed for Hodgkin lymphoma with predominantly lymph node involvement and does not take into account the extent of LTI. However, ENKTCL is exclusively an extranodal lymphoma. When used to assess localized ENKTCL, the Ann Arbor system fails to indicate the extent of LTI to guide treatment decisions. To compensate for the shortcomings of the Ann Arbor system, three ENKTCL-specified staging systems have been proposed.

Kim et al. proposed a Korea staging system in 2009 to classify ENKTCL into limited disease and extensive disease (141). However, for extensive disease, the prognosis and treatment strategies for Ann Arbor stage I/II disease with LTI and disseminated disease are obviously different. Further, for limited disease, the prognosis of Ann Arbor stage I disease is obviously different from that of Ann Arbor stage II disease. Given the solid cancer features of ENKTCL, we previously proposed a TNM (Tumor-Node-Metastasis) staging system (7). Obviously, locoregional disease is better stratified because this staging system is based solely on the extent of anatomical structures involved by the lymphoma. However, due to the complex anatomy of the UAT, this TNM staging system is too complex and inconvenient to use. Recently, the Chinese Southwest Oncology Group and the Asia Lymphoma Study Group (CA) proposed a staging method called the CA system. This method classifies Ann Arbor stage I disease into stage I or II based on the presence or absence of LTI, while stage III in CA system was identical to stage II in Ann Arbor system (142). A retrospective study comparing the CA system and the Ann Arbor system in 205 patients found that the CA system had better prognostic value than the Ann Arbor system (145). The CA system fully considers the prognostic value of both LTI and regional lymph node metastasis. Also, it is easy to use. However, CA stage II still includes a group of heterogeneous disease, as the extent of TLI varies greatly between patients, ranging from minimal to extensive infiltration around the UAT. Overall, among these staging methods (**Table 2**), the CA system is currently preferable, but the optimal staging method remains to be determined.

**Table 2:** Prospective randomized controlled studies in ENKTCL.

Author	Disease stage	Treatment modality	Arm	N	CR rate (%)	PFS (%)	OS (%)
Ma et al., 2009 (93)	I/II	CT-RT	CEOP	38	4CC: 21.1; ET: 94.4	2y: 65.8	2y: 73.3
			CEOP + semustine	37	4CC: 27.1; ET: 87.1	2y: 62.2	2y: 62.2
Wei et al., 2020 (69)	I/II	CT-RT-CT	SVILE	36	3CC: 38.9; ET: 83.4	3y: 88.3	3y: 88.8
			P-GemOx	33	3CC: 39.4; ET: 97.0	3y: 93.9	3y: 97.0
Zhang et al., 2021 (77)	I/II	RT alone	RT	35	ET: 48.6	5y: 56.5	5y: 60.4
		CT-RT	DDGP	30	ET: 73.3	5y: 82.9	5y: 85.7
Chai et al., 2022 (75)	I/II	RT-CT	GDP + chidamide	37	ET: 83.8	2y: 75.2; 5y: 67.5	2y: 89.2; 5y: 89.2
			GDP	37	ET: 78.4	2y: 70.2; 5y: 66.7	2y: 83.8; 5y: 81.0
Huang et al., 2019 (143)^a^	I-IV	CT-RT	P-GemOx + thalidomide	85	4CC: 60.0	3-y: 61.4^b^	3-y: 63.4^b^
			AspMetDex	80	4CC: 55.0		
Wei et al., 2020 (69)	III/IV	CT alone	SVILE	16	3CC: 6.2; ET: 68.8	3y: 46.2	3y: 68.8
			P-GemOx	18	3CC: 11.1; ET: 61.1	3y: 65.7	3y: 72.2
Wang et al., 2022 (130)	III/IV ^c^	CT alone	DDGP	40	ET: 67.5	3y: 56.5	5y: 74.3
			SMILE	40	ET: 47.5	3y: 41.8	5y: 51.7

CR, complete response; PFS, progression-free survival; OS, overall survival; CT, chemotherapy; RT, radiotherapy; CEOP, cyclophosphamide, vincristine, etoposide, and prednisone; CC, cycles of chemotherapy; ET, end of treatment; SVILE, ifosfamide, PEG-Asp, vindesine, etoposide, and dexamethasone; P-GemOx, PEG-Asp, gemcitabine, and oxaliplatin; DDGP, PEG-Asp, gemcitabine, cisplatin and dexamethasone; GDP, gemcitabine, cisplatin, and dexamethasone; AspMetDex, PEG-Asp, methotrexate, and dexamethasone; SMILE, dexamethasone, methotrexate, ifosfamide, L-asparaginase, and etoposide.

a This was a multicenter randomized phase 2 trial. The analysis included 107 patents with newly diagnosed early-stage disease and 58 patients with advanced-stage or R/R disease. The CR rates were from patients with both early-stage and advanced-stage disease;

b The survival data were for the whole cohort. Separate data for each arm were not reported;

c This study adopted the CA staging system and included Ann Arbor stage II disease. PEG-Asp was used in the DDGP arm, while L-Asp in the SMILE arm.

## 6 Treatment of early-stage ENKTCL

### 6.1 RT

The current National Comprehensive Cancer Network (NCCN) guidelines recommend combined chemoradiotherapy (CRT) for fit patients and RT alone for unfit patients with early-stage nasal ENKTCL. RT has an essential role in improved OS and PFS in patients with early-stage nasal ENKTCL (88, 146, 147). The 5-year OS rate in patients with early-stage disease treated with CT alone is only 12% to 45% (**Figure 4**) (4, 31, 32, 51, 59, 88, 91, 148). Emphatically, RT can’t be omitted in patients who have achieved CR after induction CT. According to a multicenter study by the China Lymphoma Collaborative Group (CLCG), for Ann Arbor stage I/II ENKTCL patients who reached CR after Asp-based CT, the 5-year OS was 84.9% and 58.9% in patients with and without RT, respectively (149).

During the early decades, patients receiving RT often had unsatisfactory outcomes due to the use of suboptimal radiation doses (34, 88). Patient outcomes did not improve significantly until the 1970s with the use of a higher dose of 50 Gy, which is recognized necessary to achieve long-term survival (88, 150). Modern RT for ENKTCL (including risk-adapted therapy, target volume, and dose guidelines) has been elaborately described by Qi et al. (151).

### 6.2 CT

In the Anth era, the 5-year OS was 47.3-83% in Ann Arbor stage I/II patients receiving RT alone (31–34, 37, 152–154) and 37.9-76% in those receiving combined CRT (29, 30, 32, 33, 37, 40, 46). There was no convincing evidence that adding CT to RT resulted in additional survival benefit (28, 30, 32, 33, 37, 40, 44, 152, 155, 156). However, this status has changed after the introduction of Asp. A randomized controlled study and a large cohort retrospective study both demonstrated the benefits of adding Asp-based CT. Zhang et al. randomized 65 patients with early-stage ENKTCL into RT alone group or DDGP (cisplatin, dexamethasone, PEG-Asp, and gemcitabine) followed by RT group. The 5-year PFS (56.5% *vs*. 82.9%, *P* = 0.023) and OS (60.4% *vs*. 85.7%, *P* = 0.040) were significant higher in the combined CRT group (**Table 1**) (77). Nevertheless, not all patients with early-stage disease can benefit from the addition of Asp-based CT. Zheng et al. evaluated the survival benefit of Asp-based *vs*. non-Asp-based CT plus RT in a retrospective cohort of 376 patients with early-stage ENKTCL. They stratified patients into low-, intermediate- and high-risk groups based on 5 clinical parameters (age > 60 years, stage II, elevated lactate dehydrogenase, poor performance status, and LTI), and found that Asp-based CT significantly improved 5-year OS in intermediate- and high-risk patients (84.4% *vs*. 74.5%, *P* = 0.014) (157). How to select patients who can benefit from the addition of Asp-based CT needs to be defined in randomized controlled studies. The survival trends in patient with early-stage ENKTCL treated with different types of CT are showed in **Figure 5**.

Another unanswered issue about CT is that how many CT courses are needed for early-stage ENKTCL. As shown in **Table 3**, most studies used 3 to 6 cycles of CT, and there is no evidence that short-course CT is inferior to long-course CT. A short-course of two cycles of modified SMILE CT and sequential RT in 18 cases with early-stage ENKTCL at the Memorial Sloan Kettering Cancer Center showed a post-CT CR rate of 67% and a 5-year OS of 83.3% (162). The outcomes were similar to studies with long-course CT. The optimal number of CT courses needs to be determined in randomized controlled studies.

**Table 3:** Single arm prospective studies in NKTCL.

Author	Disease status	N	Asp	CT regimen used	Treatment modality	Courses of CT	CR rate (%)	PFS (%)	OS (%)
Lee et al., 2006 (35)	Stage I/II	16		IMEP	CT-RT	6	6CC: 69.0; ET: 81	–	3y: 80.4
Kim et al., 2009 (42)	Stage I/II	30		VIPD	CCRT-CT	Weekly DDP + 3	ET: 80	3y: 85.2	3y: 86.3
Yamaguchi et al., 2009 (95, 96)	Stage I/II	33		DeVIC	CCRT	3	ET: 75.0	2y: 67.0 5y: 67.0	2y: 78.0 5y: 73.0
Jiang et al., 2012 (45, 97)	Stage I/II	26	L-Asp	LVP	CT-RT-CT	2 + (2–4)	2CC: 42.3; ET: 80.8	2y: 80.6 5y: 64.0	2y: 88.5 5y: 64.0
Aviles et al., 2013 (51)	Stage I/II	202		CMED	RT-CT	6	ET: 91.0	–	5y: 86.0
Lin et al., 2013 (49)	Stage I/II	31	L-Asp	CHOP	CT-RT	6	4CC: 71.5; ET: 81.6	2y: 81.0	2y: 80.1
Wang et al., 2013 (50)	Stage I/II	27	L/P-Asp	GELOX	CT-RT	2 + 4	2CC: 55.6; ET: 74.1	2y: 86.0	2y: 86.0
Ke et al., 2014 (52)	Stage I/II	32		GDP	CCRT-CT	Weekly DDP + 3	ET: 84.4	3y: 84.4	3y: 87.5
Kim et al., 2014 (54)	Stage I/II	44		IMEP	CT-RT	6	6CC: 27.0; ET: 67.0	3y: 56.0	3y: 66.0
Kim et al., 2014 (53)	Stage I/II	30	L-Asp	IMEP	CCRT-CT	weekly DDP + 2	ET: 87.0	5y: 60.0	5y: 73.0
Tsai et al., 2015 (55)	Stage I/II	33		DVIP	CCRT-CT	2 DEP + 2	ET: 42.0	2y: 64.0 5y: 60.0	2y: 73.0 5y: 66.0
Yoon et al., 2016 (61)	Stage I/II	28	L-Asp	IMEP	CCRT-CT	Weekly DDP + 2	ET: 82.1	3y: 74.1	3y: 81.5
Jiang et al., 2017 (62)	Stage I/II	66	L-Asp	DEP	CT-CCRT-CT	2 + 2 DDP + 2	ET: 83.3	3y: 67.4	3y: 70.1
Xu et al., 2017 (63)	Stage I/II	40	P-Asp	MESA	CT-RT-CT	2 + 2	2CC: 71.1; ET: 89.5	2y: 89.1	2y: 92.0
Qi et al., 2018 (64)	Stage I/II	40		GDP	RT-CT	4	ET: 95.0	2y: 84.7 5y: 79.4	2y: 89.9 5y: 82.1
Zheng et al., 2018 (65)	Stage I/II	21	P-Asp	CHOP	RT-CT	6	ET: 90.5	–	3y: 80.5
Liu et al., 2020 (66)	Stage I/II	30	P-Asp	DICE	RT-CT	3	ET: 96.7	5y: 86.0	5y: 87.0
Wang et al., 2020 (67)	Stage I/II	30	P-Asp	P-Asp alone	CCRT-CT	2 + 4	ET: 100	2y: 90.9	2y: 92.8
Wei et al., 2020 (68)	Stage I/II	26	P-Asp	GDP -ML	CT-RT-CT	2 + 2	ET: 76.9	–	2y: 88.1
Zhu et al., 2020 (70)	Stage I/II	30	P-Asp	GDP	CCRT-CT	Weekly DDP + 3	ET: 93.3	5y: 89.4	5y: 93.3
Zhang et al., 2021 (71)	Stage I/II	81	L-Asp^!^	DICE	CT-RT	4	4CC: 11.1; ET: 84.0	5y: 63.4	5y: 82.4
Hu et al., 2022 (72)	Stage I/II	64	P-Asp	COEPL	CT-CCRT-CT	2 + 2 (VLP) + 2	ET: 82.0	3y: 78.1	3y: 81.2
Wang et al., 2022 (73)	Stage I/II	31	P-Asp	GAD-M	CT-RT-CT	(2–4) + (4–2)	2CC: 54.8; ET: 90.3	3y: 80.4 3y: 80.6	3y: 77.0 5y: 80.6
Zhu et al., 2022 (74)	Stage I/II	52	P-Asp	GELAD	CT-RT-CT	2 + 2	2CC: 46.2; ET: 92.3	2y: 90.4 4y: 90.4	2y: 94.2 4y: 94.2
Lee et al., 2006 (35)	Stage III/IV	8		IMEP	CT alone		13.0	–	mOS: 2.7m 3y: 30%
Kim et al., 2009 (99)	R/R	32		IMEP	CT alone		37.5	mTTF: 3.7m	mOS: 8.2m 5y: 24.8
Jaccard et al., 2011 (100)	R/R	19	L-Asp	AspMetDex	CT-RT/HSCT		61.0	mPFS: 12.2m	mOS: 12.2m
Yamaguchi et al., 2011 (101)	Stage IV/R/R	38	L-Asp	SMILE	CT-HSCT (allo/auto)		45.0	1y: 53	1y: 55
Shi et al., 2015 (158)	R/R	16		Chidamide			6.0	–	–
Zheng et al., 2018 (65)	Stage III/IV	12	P-Asp	CHOP	CT alone		50.0	–	–
Wei et al., 2020 (68)	Stage III/IV	18	P-Asp	GDP-ML	CT-HSCT (auto)		33.3	2y: 33.2	2y: 35.6
Kim et al., 2020 (159)	R/R	21		Avelumab			24.0	mPFS: 2.7m	–
Song et al., 2021 (114)	Stage III/IV	27	L-Asp	VIDL	CT-HSCT (auto)		63.0	mPFS: 13.2m	mOS: 27.0m
Hu et al., 2022 (72)	Stage III/IV	16	P-Asp	COEP	CT-HSCT (auto)		55.6	3y: 45.7	3y: 48.1
Gao et al., 2020 (160)	Asp-R	37		Sintilimab + chidamide			44.4	1y: 66.0	1y: 79.1
Huang et al., 2021 (161)	Asp-R	32		Daratumumab			0	mPFS: 53.0d	mOS: 141.0d
Tao et al., 2021 (111)	Asp-R	28		Sintilimab			21.4	–	1y: 82.1 2y: 78.6
Huang et a. 2022 (98)	Asp-R	78		GEMSTONE-201			37.2		2y: 54.6

IMEP, ifosfamide, methotrexate, etoposide, and prednisone; DDP, cisplatin; VIPD, etoposide, ifosfamide, dexamethasone, and cisplatin; DeVIC, dexamethasone, etoposide, ifosfamide, and carboplatin; LVP, L-Asp, vincristine, and prednisone; GELOX, gemcitabine, L/P-Asp, and oxaliplatin; DICE, dexamethasone, ifosfamide, etoposide, and cisplatin; DEP, dexamethasone, etoposide, and cisplatin; DVIP, dexamethasone, etoposide, ifosfamide, and cisplatin; MESA, methotrexate, etoposide, dexamethasone, and PEG-Asp; CMED, cyclophosphamide, methotrexate, etoposide, and dexamethasone; SMILE, dexamethasone, methotrexate, ifosfamide, L-asparaginase, and etoposide; AspMetDex, L-asparaginase, methotrexate, and dexamethasone; HSCT, hematopoietic stem cell transplantation; DICE, dexamethasone, ifosfamide, cisplatin, and etoposide; GDP-ML, gemcitabine, dexamethasone, cisplatin, methotrexate, and PEG-Asp; COEPL, cyclophosphamide, vincristine, etoposide, prednisone, and P-Asp; VLP, vincristine, pegaspargase, and prednisone; GAD-M, gemcitabine, PEG-Asp, dexamethasone, and methotrexate; GELAD, gemcitabine, etoposide, PEG-Asp, dexamethasone; VIDL, etoposide, ifosfamide, dexamethasone, and L-Asp; Asp-R, asparaginase-resistant. ! L-ASP was delivered on d1-4.

### 6.3 Combined modality treatment of CT and RT

There are 3 combined modalities of CT and RT for early-stage ENKTCL: sequential method (CT followed by RT, or RT followed by CT), sandwich method (CT before and after RT), and concurrent method (CT during the period of RT). There is no available evidence showing which modality is more preferable at the moment. The sequential and sandwich methods are mainly used in China, while concurrent method is mainly used in Japan and Korea. The development of sequential and sandwich modalities in the early days was based on the understanding that this aggressive tumor requires aggressive CT, which would not be tolerated when administered concurrently with RT.

In the Anth era, early disease progression frequently occurred in patients treated with induction CT due to resistance, so the importance of upfront RT was emphasized (57, 80, 163). However, increased risk of systemic relapse was also observed in patients receiving upfront RT (59). In the current Asp era, early disease progression is not a big concern as most patients are sensitive to Asp-based treatment. Two multicenter retrospective studies of large cohorts evaluated how the sequence of CT and RT affects outcomes of patients treated primarily with Asp-based CT. Based on data from 1,360 patients, the China Lymphoma Collaborative Group Study found that patients treated with CT followed by RT had similar survival to those treated with RT followed by CT (164). Kwong et al. compared sequential CRT *vs*. concurrent CRT in 303 cases and observed similar survival as well (165). The optimal sequence of CT and RT needs to be defined in randomized controlled studies.

In today’s view, aggressive CT is unnecessary for ENKTCL. Mild regimens, such as P-GemOx (GELOX) and DDGP, have equal or better efficacy and a more favorable safety profile than aggressive regimens (50, 69, 70, 130, 143). Given the trend towards more simplified CT, the feasibility of concurrent CRT should be reevaluated. Recent studies have showed that Asp alone or Asp combined with immunotherapy also yielded excellent efficacy in patients with early-stage ENKTCL when combined with RT (27, 67). These studies, although using small sample sizes, suggest that more simplified, more effective, and less toxic regimens are feasible.

### 6.4 Anti-PD-1/PD-L1 immunotherapy

Given the high activity in advanced-stage ENKTCL, anti-PD-1/PD-L1 immunotherapy is being tried in early-stage disease. Sun et al. recently reported the results of a retrospective study of early-stage ENKTCL treated with PEG-Asp combined with a PD-1 inhibitor, anlotinib (a multi-tyrosine kinase inhibitor approved in China for lung cancer), and sequential RT. Patient outcomes were excellent, with 100% 3-year PFS and OS, but a limitation of the study was that only 8 patients were included (27). The role of anlotinib in this combination was unknown, but the combination of the two most powerful ENKTCL drugs (PEG-Asp and PD-1 inhibitor) deserves further investigation in more patients. A phase 2 trial of this regimen is pending (NCT03936452), and a phase 3 randomized controlled trial of PEG-Asp-based CRT with or without PD-1 blockade therapy in early-stage ENKTCL is ongoing (NCT04365036). Several studies are currently underway to examine the efficacy of more simplified immunotherapy-based treatment approaches in patients with early-stage ENKTCL: PD-1 inhibitor and PEG-Asp combined with RT (NCT04676789), PD-1 inhibitor, PEG-Asp, and chidamide combined with RT (NCT04414969), PD-1 inhibitor monotherapy (NCT03728972), and PD-1 inhibitor concurrently with RT (NCT04417166, NCT05477264, and NCT05149170).

## 7 Treatment of advanced-stage and R/R ENKTCL

### 7.1 CT

The survival of patients with advanced-stage ENKTCL is extremely poor. The current NCCN guidelines recommend Asp-based CT for advanced-staged disease. As shown in **Figure 3**, the CR rate of patients treated with Asp-absent CT (including Anth- and non-Anth-based CT) ranged from 13.0% to 37.5%, and the 5-year OS ranged from 16.7% to 30.0%. The efficacy of Asp-based CT is better than that of Asp-absent CT, but it is still unsatisfactory. In a prospective cohort study of the International T-cell Project, including 166 patients from 40 centers in 14 countries across four continents (Asia, Europe, North America, and South America), the median OS was only 10 months (4). As in early-stage disease, aggressive CT in advanced-stage disease may result in greater toxicity rather than greater efficacy (166). The median OS was several months as well in patients treated with SMILE or modified SMILE regimen and hematopoietic stem cell transplantation (HSCT) (162, 167). Patients with relapsed disease after front-line treatment had even worse survival. A multicenter retrospective study analyzing 179 patients with R/R ENKTCL from four countries found a median second OS of 6.4 months after relapse (168). Patients with extranasal ENKTCL had similar survival to those with advanced-stage nasal ENKTCL. The median OS was 2.83 to 9 months for gastrointestinal ENKTCL (169–172), 9.5 months for testicular disease (173), and 15.5 to 29 months for cutaneous ENKTCL (174, 175). These data indicate that extranasal ENKTCL should be managed like advanced-stage nasal ENKTCL.

### 7.2 HSCT

The role of HSCT in the treatment of ENKTCL is controversial. Auto-HSCT did not lead to better outcomes compared to Asp-based CT (176, 177). Several small cohort retrospective studies have evaluated the efficacy of allo-HSCT in patients with advanced-stage ENKTCL. A Japanese study of 28 patients (22 with NKTCL, 3 with blastic NK-cell lymphoma, and 3 with aggressive NK-cell leukemia) concluded that 2-year PFS and OS were 34% and 40%, respectively (178). In another study of 12 patients, 7 cases survived in remission with a median follow-up of 13 months (179). After investigating 18 patients with advanced-stage or R/R ENKTCL who underwent allo-HSCT, a multicenter study by the Asia Lymphoma Study Group observed 5-year PFS and OS of 51% and 57%, respectively (180). In a non-Asian cohort with 27 patients undergoing HSCT (14 with auto-HSCT and 13 with allo-HSCT), the 3-year OS was 64% for consolidative auto-HSCT in first-line treatment and 39% for allo-HSCT in salvage treatment (181). These data seem encouraging, but there was patient selection bias in these studies, as patients who received HSCT generally had favorable performance status and good response to previous treatment. Besides, it may not be better than Asp-based CT (**Figure 3**). There is no doubt that HSCT will move to late-line treatment with the increasing number of novel drugs today.

### 7.3 RT

Given the sensitivity of ENKTCL to RT, RT is used in selected patients with advanced-stage disease. According to the International T-cell Project, patients with advanced-stage disease receiving combined CRT had significantly better survival than those receiving CT alone (3-year OS, 66% *vs*. 24%; 5-year OS, 58% *vs*. 24%; both Ps < 0.001) (4). However, there was clearly a selection bias. Patients who received RT inevitably had lower tumor burden and better response to induction CT than those who did not receive RT. Nevertheless, it is suggested that RT can be delivered to selected patients with oligometastases.

### 7.4 Anti-PD-1/PD-L1 immunotherapy

The positive rate of PD-L1 in ENKTCL cells is 39-100% (8). Overexpression of PD-L1 induced by EBV infection, on the one hand, is a potential mechanism by which ENKTCL avoids immune surveillance (182, 183), on the other hand, is a predictor of favorable response to PD-1 blockade treatment (159, 160). Kwong et al. first reported that PD-1 blockade was highly active in R/R ENKTCL in a series of 7 cases (26). Its outstanding efficacy was subsequently confirmed in clinical trials. In a phase 2 study, 28 patients with Asp-resistant ENKTCL were treated with the PD-1 inhibitor sintilimab. The CR rate and objective response rate (ORR) were 21.4% and 75%, respectively, and the 2-year OS was 78.6% (111). In another phase 2 trial of 78 cases with Asp-resistant ENKTCL treated with the anti-PD-L1 antibody sugemalimab, the CR was 37.2% and the 2-year OS was 54.6% (98). Clearly, the median second OS was > 2 years in both trials. The current NCCN guidelines recommend anti-PD-1 immunotherapy for Asp-resistant R/R ENKTCL. Promising results have also been observed in patients treated with immunotherapy combined with small molecule targeted drugs, such as histone deacetylase inhibitors (HDACi) (160, 184, 185). The combination of PD-1 inhibitor and HDACi chidamide was evaluated in a prospective phase Ib/II study in patients with Asp-resistant ENKTCL. The preliminary analysis of 37 cases showed that the CR rate, 1-year PFS, and OS was 44.4%, 66.0%, and 79.1%, respectively (160). The combined treatment showed activity even in patients with immunotherapy-resistant lymphoma, as HDACi also has immunomodulatory effects beyond anticancer effect (185, 186). Clinical trials investigating the efficacy of PD-1 blockade combined with a DNA demethylating inhibitor (DNMTi) (NCT04279379) or anti-CD38 antibody (NCT04763616) in R/R ENKTCL are ongoing.

Due to its high activity, immunotherapy has recently been used in the front-line treatment of advanced-stage disease. Cai et al. treated 9 newly diagnosed stage III/IV ENKTCL cases using a PD-1 inhibitor in combination with the P-GemOx regimen. The CR rate, 1-year PFS, and OS was 77.8%, 66.7%, and 100%, respectively (109). Although a small cohort retrospective study, it suggests that the combination of the two most powerful drugs (PEG-Asp and PD-1 blockade) may be a good option for front-line therapy of advanced-stage ENKTCL, and that aggressive CT may not be necessary in the modern era. Additional results of a prospective study (NCT04127227) of this combination treatment are pending. A more simplified regimen combing PD-1 blockade and PEG-Asp is currently being tested in prospective trials (NCT04096690 and NCT04004572) in patients with newly diagnosed advanced-stage ENKTCL.

### 7.5 Other immunotherapies

ENKTCL patients are universally EBV positive, making the viral protein latent membrane proteins (LMP) interesting treatment targets. LMP-targeted therapy has shown encouraging activity in several studies. In a phase 2 study, Kim et al. created autologous EBV-specific T cells in 47 patients with advanced-stage or R/R ENKTCL. The CR rate was 30% in 10 R/R patients receiving the cell therapy (187). Bollard et al. treated 29 patients with EBV-related lymphoma using LMP-specific cytotoxic T lymphocytes (CTL). The results showed that 11 of 21 patients with R/R disease achieved CR at the time of CTL infusion (188). Cho et al. treated 13 patients (11 early-stage patients and 2 advanced-stage patients) with EBV LMP-specific CTL cells when CR was achieved following CT, RT, and/or HSCT. The 4-year OS and PFS were 100% and 90%, respectively (189). Ando et al. developed an EBV-induced pluripotent stem cell (iPSC)-derived LMP-specific CTL that exhibited robust ENKTCL suppressive effects *in vitro* and *in vivo* (190). More clinical trials of EBV-targeted T cell therapy are underway (NCT03789617 and NCT03671850).

A subset of ENKTCL cells express CD30 and CD38, both of which have available targeted drugs. The anti-CD30 antibody conjugate brentuximab vedotin (BV) has been reported to be effective in individual cases (191, 192). In a BV phase 2 study of 33 patients with R/R CD30-positive non-Hodgkin lymphoma, 7 had ENKTCL. One ENKTCL patient achieved CR and another ENKTCL patient achieved partial response (PR) (193). The current NCCN guidelines recommend BV monotherapy for Asp-resistant R/R ENKTCL. The combination of BV with PD-1 inhibition (NCT05316246) and anti-CD30 CAR-T therapy (NCT04952584, NCT04008394, and NCT04526834) are currently under investigation in patients with CD30+ R/R lymphocyte malignancies. The anti-CD38 antibody daratumumab has been examined in a phase 2 study in patients with R/R ENKTCL with an ORR of 25% and a median PFS of 53 days (161) (**Table 3**). Zhang et al. screened a panel of biomarkers (B7-H3, CD70, TIM-3, VISTA, ICAM-1, and PD-1) in NKTCL cell lines and found that B7-H3 (CD276) was highly and homogeneously expressed in these cells. Therefore, they constructed a novel anti-B7-H3/CD3 bispecific T-cell engaging antibody and B7-H3-redirected CAR-T cells, both of which showed antitumor activity *in vitro* and *in vivo* (194). CD7 is positive in 84.8% of patients with ENKTCL. In a recently reported phase 1 stage study of 20 cases with CD7-positive R/R T-ALL or T-cell lymphoblastic lymphoma, 19 patients achieved bone marrow CR and 5 achieved extramedullary CR (195). A prospective trial of anti-CD7 CAR-T cell therapy in CD7-positive T-cell lymphoma (including ENKTCL) is ongoing (NCT04004637). ENKTCL is an IL-2-dependent cancer (196). The binding of IL-2 to IL2α (CD25) exerts pro-inflammatory effects and promotes lymphomagenesis and drug resistance (197, 198). Therefore, blocking IL2-CD25 binding is a potential therapeutic strategy. Wang et al. have launched a prospective trial after reporting a R/R ENKTCL patient who responded well to anti-CD25 antibody therapy (NCT04337593).

Among these immunotherapies under investigation, anti-LMP CAR-T therapy or EBV-specific T cell therapy might be the most promising treatment.

### 7.6 Small molecule drugs

**Figure 7:**
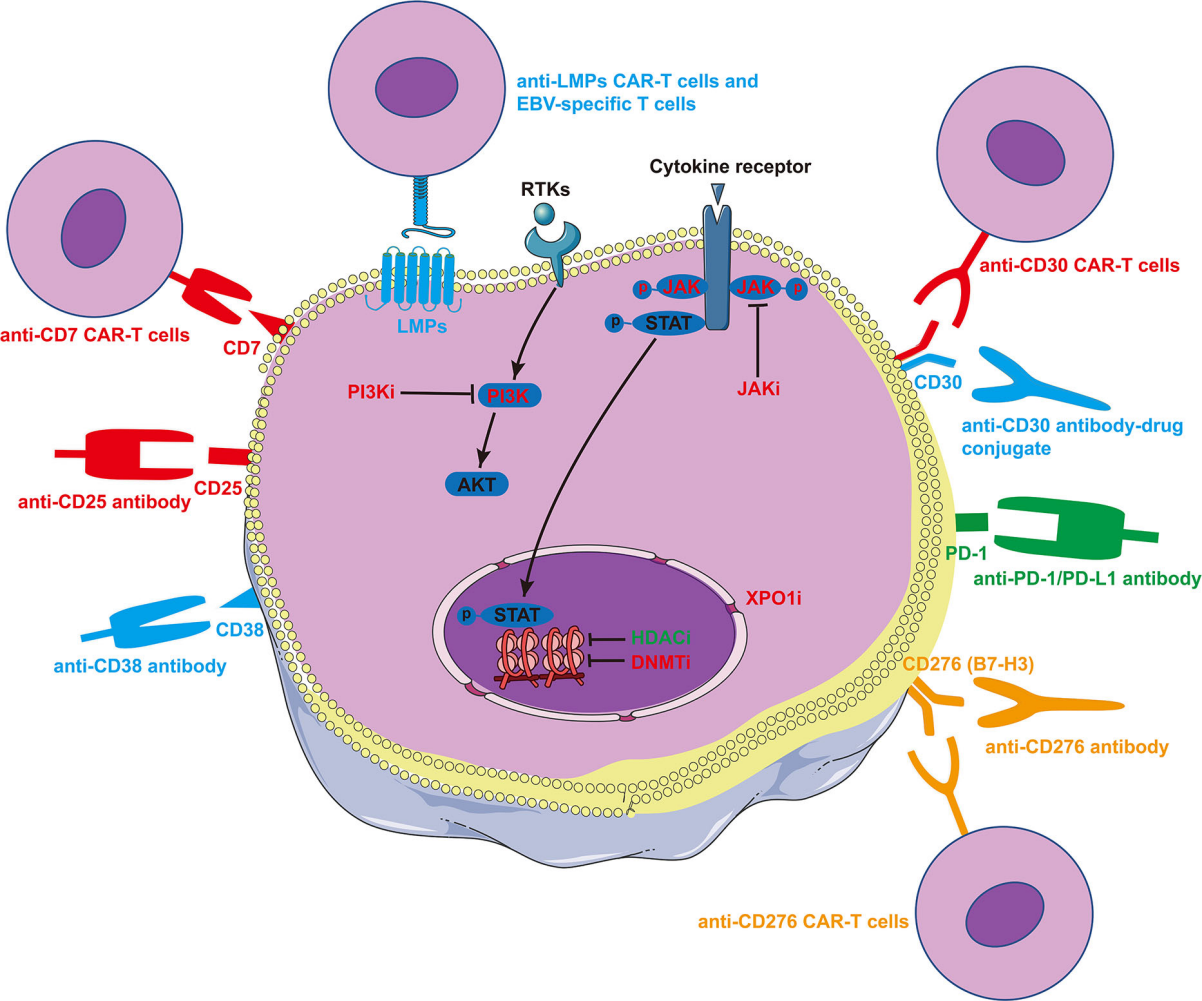
Schematic illustration of targeted therapies in ENKTCL. Green indicates that the targeted therapies have been widely used in practice; cyan indicates that the therapies are being investigated in early-stage clinical trials; orange indicates that the therapies have shown activity in animal models; and red indicates that the therapies are currently under investigation in clinical trials.

Epigenetic modifiers, including *MLL2*, *MLL3*, *BCOR*, *TET2*, *EP300*, and *ARID1A*, are mutated in about 24.8% of cases with ENKTCL (9). Accordingly, epigenetic regulation is an important treatment strategy for this disease. The HDACi chidamide has been approved in China for the treatment of R/R PTCL, including ENKTCL. In a phase 2 study of chidamide in patients with R/R PTCL, the CR rate was 6% in 16 patients with ENKTCL (158). When two additional real world studies were included, the pooled CR rate was 16% (ORR 38%), involving a total of 115 R/R ENKTCL patients (158, 199, 200). Romidepsin and belinostat are HDAC inhibitors used in western countries. The current NCCN guidelines recommend HDACi for Asp-resistant R/R ENKTCL. The DNMTi azacytidine and decitabine are being actively tested in combination treatment in patients with R/R ENKTCL (NCT04899414 and NCT04279379). Moreover, common genetic aberrations (18.2%) in ENKTCL involve genes related to the JAK-STAT pathway (*JAK3*, *JAK2*, *STAT3*, *STAT5B*, *SOCS1*, and *PTPRK*). *JAK* or *STAT* activating mutations lead to constitutive activation of the JAK-SATAT pathway, which plays a major role in ENKTCL cell growth and survival (9, 201). These findings make JAK an interesting target for the treatment of ENKTC. The efficacy of JAK inhibitor in R/R T- and NK-cell lymphoma is currently under investigation (NCT02974647). Mutations in genes associated with the RAS-MAPK pathway (*MAP3K*s, *BRAF*, and *EPH1A*) occur in 14.4% of ENKTCL cases (9). Alisertib, which targets aurora kinase A, a downstream molecule of MAPK, has been used in 5 cases of R/R ENKTCL as part of two studies, but only one patient achieved PR (202, 203). XPO1 is a promising target for the treatment of both hematological and non-hematological cancers (204, 205). Preliminary results from a phase Ib study of the XPO1 inhibitor ATG-010 plus CT in heavily pretreated patients with R/R PTCL and ENKTCL have been reported at the 2021 ASH meeting. ORR and CR were observed in 3 and 2 of the 5 ENKTCL cases analyzed, respectively (206). The final results of this trial are pending (NCT04425070). PI3K inhibitors (PI3Ki) have demonstrated encouraging activity in R/R PTCL patients in multiple trials (207–209). Currently, several trials are assessing the efficacy and safety of PI3Ki combined with HDACi or other agents in patient with R/R PTCL, including ENKTCL (NCT05083208, NCT04774068, and NCT04639843). The above-mentioned targeted therapies are illustrated in **Figure 7**. In addition, deregulation of apoptosis through *TP53* mutations provides a further growth advantage for this disease (210). Targeting the TP53-MDM2 interaction is also a potential treatment approach. MDM2 inhibitors are currently being tested intensely in various cancers, including lymphomas (211). To date, small molecule drugs, including HDACi, JAK inhibitor, aurora kinase A inhibitor, only demonstrated mild efficacy in T/NK-cell lymphomas. PI3Ki showed higher response rates than these drugs, but only limited data exist and long-term efficacy still need to be observed. At present and in the near future, these drugs might be choices in late line treatment or as partner drugs in combined treatment for ENKTCL.

## 8 Biomarkers for treatment decision making

### 8.1 Predictive markers

Biomarker-driven individualized and precision treatment is the way toward better efficacy, lower toxicity, and higher cost-performance. The genetic landscapes of ENKTCL have been described in several studies with relatively large sample sizes in recent years. *DDX3X*, *TP53*, *BCOR*, *STAT3*, and *MLL2* are the most commonly mutated genes that act as tumor suppressors or epigenetic modifiers or participate in signaling pathways (JAK-STAT, NF-κB, and RAS-MAPK) (8, 212–215). *TP53* mutation was identified as an adverse prognosticator in two studies (212, 214) and *DDX3X* in one study (212), but the predictive value of these genes was inconsistently reported in others (213, 215).

*PD-L1* rearrangement is a marker of response to PD-1 blockade treatment. Lim et al. sequenced tumor samples from 19 R/R ENKTCL patients treated with the PD-1 inhibitor pembrolizumab. All 4 cases with complete and durable responses harbored *PD-L1* rearrangement, while all 10 non-responders were wild-type *PD-L1*. Clearly, *PD-L1* rearrangement is 100% specific in identifying responders to immunotherapy. In this study, PD-L1 expression levels were not associated with clinical response (216). However, higher PD-L1 expression levels did correlate with better immunotherapy response in two prospective studies (159, 160). The predictive value of *PD-L1* rearrangement on clinical response needs to be verified in more patients.

### 8.2 Molecular subtypes of ENKTCL

Molecular subtyping may help identify patients more likely to benefit from a specific treatment. The following three studies explored the molecular subtyping of ENKTCL and are summarized in **Table 4**. Xiong et al. proposed a molecular subtyping scheme based on an integrated analysis of the genomic and transcriptomic features of ENKTCL. Patients were classified into three subtypes: HEA subtype, enriched in T cell gene expression and mutations in epigenetic regulators (*EP300*, *HDAC9*, and *ARID1A*); TSIM subtype, characterized by aberrations in tumor suppressors (*TP53* mutation and del6q21) and immune modulators (JAK-STAT mutation/amplification and amp9p24.1/PD-L1/L2 locus); and MB subtype, enriched in *MYC*-associated aberrations, mainly *MGA* mutations, and loss of heterozygosity (LOH) at the *BRDT* locus. This subtyping scheme has prognostic value in patients with advanced-stage disease and may have value in individualized treatment decision-making (213). Dong et al. classified ENKTCL as C1 to C7 based on genome-wide mutation and genomic copy number alteration analysis. According to this classification, patients in C5 and C7 have a good prognosis, while patients in C6 have the worst prognosis (214). Lim et al. generated a genomic prognostic model (GPM) based on next-generation sequence of 260 ENKTCL tumors in which mutations in 13 genes (*BCOR*, *JAK3*, *KRAS*, *MYH11*, *DCC*, *ITK*, *NOTCH1*, *FAS*, *RET*, *BIRC3*, *MLLT1*, *LRP1B*, and *NRG1*) were associated with poorer survival. The representative genomic alterations and prognostic values of subtypes vary largely among these studies. This discrepancy is at least partially attributable to the inclusion of patients with heterogeneous disease status (mixed early-stage and advanced-stage disease) and treatments. Future subtyping studies should be conducted in patients receiving homogeneous treatment.

**Table 4:** Molecular subtyping schemes of ENKTCL published in recent years.

Author	Subtype	Representative genomic alterations or immune characteristics	Prognosis	Potentially effective treatment
Xiong et al. (213)	HEA	Mutations in *HDAC9*, *EP300*, and *ARID1A*	Good	HDAC inhibitor
	TSIM	Mutations in JAK-STAT pathway and *TP53*, amp*JAK2* locus; amp17q21.2/*STAT3*/*5B*/*5A* locus, amp9p24.1/*PD-L1/2* locus, del6q21	Intermediate	PD-1 blockade
	MB	*MGA* mutation, 1p22.1/*BRDT* LOH	Poor	MYC inhibition
Dong et al. (214)	C1	Higher CN complexity including gains and losses of 17q21(*STAT3*), 8q24(*MYC*), and del19q	Intermediate	-
	C2	*KMT2D* mutation and chr2 gain	Intermediate	-
	C3	*NOTCH2* mutation and del17p	Intermediate	-
	C4	*DDX3X* mutation and del1p36	Intermediate	-
	C5	CN gain of chr19q/q13 and *JAK3* gain	Good	-
	C6	Aberrations in RAS/RAF/MAPK pathway, *JAK3*, *BCOR*, and *TP53*	Poor	-
	C7	*TET2* loss and *ARID1B* mutation	Good	-
Lim et al. (215)	GPM	Mutations in *BCOR*, *JAK3*, *KRAS*, *MYH11*, *DCC*, *ITK*, *NOTCH1*, *FAS*, *RET*, *BIRC3*, *MLLT1*, *LRP1B*, *NRG1*	Poor	-
Cho et al. (217)	Immune tolerance	High-Treg counts (> 500/HPF)	Good	Good response to PD-1 blockage
	Immune evasion-A	High cytotoxic T-cell counts, high PD-L1 expression, low Treg counts (PD-L1 > 10%)	Intermediate	Intermediate response to PD-1 blockage
	Immune evasion-B	Not otherwise specified	Intermediate	Intermediate response to PD-1 blockage
	Immune silenced	Immune response exhausted (Process-type CD68 > 90%)	Poor	Poor to PD-1 blockage

In addition, Cho et al. developed an immune subtyping model that classifies ENKTCL into four tumor immune microenvironment subgroups using three immunohistochemical markers, FOXP3, PD-L1, and CD68. The four subgroups were named immune tolerance, immune evasion-A, immune evasion-B, and immune silenced. The response rate to pembrolizumab was 100% (1/1) in the immune tolerance group, 60% (3/5) in the immune evasion group, and 0 (0/5) in the immune-silenced group (217). This subtyping method may guide immunotherapy but needs to be verified in more patients.

## 9 Conclusions

Survival of ENKTCL patients has improved significantly in the past two decades, mainly due to the great advances in RT technology and the introduction of Asp and anti-PD-/PD-L1 immunotherapy. RT is essential for early-stage disease. Asp-based CT benefits a proportion of patients with early-stage disease. Asp and PD-1/PD-L1 inhibitors are milestones in advanced-stage disease treatment. Their combination can further improve patient outcomes. The efficacy of Asp could be optimized by individualized administration. In the treatment of both early-stage and advanced-stage diseases, there is a trend toward more simplified regimens, less toxicity, and higher efficacy. Relevant clinical trials are currently underway. Small molecule inhibitors, monoclonal antibodies, and manufactured T cell therapies are under intensive investigation. EBV-targeted T cell therapies might be the most promising new treatment in the near future. In the future, there is no need to spend more resources comparing different combinations of Asp with cytotoxic agents. Instead, more efforts should be made to optimize the use of Asp and immunotherapy to maximize efficacy and minimize toxicity, explore ways to overcome resistance to Asp and immunotherapy, determine the optimal combination therapy of CT and RT, identify novel treatment targets, and define subpopulations who may benefit more from specific treatments.

## Author contributions

ZY, ZHY and YL: conceptualization and methodology. ZY and SY: data curation and formal analysis. ZW and WZ: software and visualization. ZY: original draft. YL and ZHY: supervision, project administration, review and editing. All authors contributed to the article and approved the submitted version.
